# Structural similarity of human papillomavirus E4 and polyomaviral VP4 exhibited by genomic analysis of the common kestrel (*Falco tinnunculus*) polyomavirus

**DOI:** 10.1007/s11259-023-10210-1

**Published:** 2023-09-09

**Authors:** Enikő Fehér, Eszter Kaszab, János András Mótyán, Dóra Máté, Krisztina Bali, Márton Hoitsy, Endre Sós, Ferenc Jakab, Krisztián Bányai

**Affiliations:** 1 HUN-REN Veterinary Medical Research Institute, Budapest, Hungary; 2 National Laboratory for Infectious Animal Diseases, Antimicrobial Resistance, Veterinary Public Health and Food Chain Safety, Budapest, Hungary; 3 National Laboratory of Virology, Szentágothai Research Centre, University of Pécs, Pécs, Hungary; 4 Institute of Metagenomics, University of Debrecen, Debrecen, Hungary; 5 Department of Biochemistry and Molecular Biology, Faculty of Medicine, University of Debrecen, Debrecen, Hungary; 6 Conservation and Veterinary Services, Budapest Zoo and Botanical Garden, Budapest, Hungary; 7 Department of Exotic Animal and Wildlife Medicine, University of Veterinary Medicine, Budapest, Hungary; 8 Department of Pharmacology and Toxicology, University of Veterinary Medicine, Budapest, Hungary

**Keywords:** Genome, Polyomavirus, Bird, VP4, Papillomavirus, E4

## Abstract

Polyomaviruses are widely distributed viruses of birds that may induce developmental deformities and internal organ disorders primarily in nestlings. In this study, polyomavirus sequence was detected in kidney and liver samples of a common kestrel (*Falco tinnunculus*) that succumbed at a rescue station in Hungary. The amplified 5025 nucleotide (nt) long genome contained the early (large and small T antigen, LTA and STA) and late (viral proteins, VP1, VP2, VP3) open reading frames (ORFs) typical for polyomaviruses. One of the additional putative ORFs (named VP4) showed identical localization with the VP4 and ORF-X of gammapolyomaviruses, but putative splicing sites could not be found in its sequence. Interestingly, the predicted 123 amino acid (aa) long protein sequence showed the highest similarity with human papillomavirus E4 early proteins in respect of the aa distribution and motif arrangement implying similar functions. The LTA of the kestrel polyomavirus shared <59.2% nt and aa pairwise identity with the LTA sequence of other polyomaviruses and formed a separated branch in the phylogenetic tree among gammapolyomaviruses. Accordingly, the kestrel polyomavirus may be the first member of a novel species within the *Gammapolyomavirus* genus, tentatively named *Gammapolyomavirus faltin*.

**Supplementary Information:**

The online version contains supplementary material available at 10.1007/s11259-023-10210-1.

Polyomaviruses are widely distributed viruses described in vertebrates (mammals, birds, fish) and in arachnids (scorpions) (Calvignac-Spencer et al. 2016; Ehlers et al. 2019; Fehér et al. 2022; Kaszab et al. 2021a; Moens et al. 2017; Schmidlin et al. 2021). The *Gammapolyomavirus* genus of *Polyomaviridae* family includes avian polyomaviruses that may cause fatal illnesses in healthy, primarily young birds (Johne and Müller 2007; Moens et al. 2017) (https://ictv.global/). Via its tropism to endothelial cells, goose hemorrhagic polyomavirus (GHPV) induces hemorrhagic diseases in goslings. Budgerigar fledgling disease virus (BFDV) infection leads to characteristic feather and beak malformations, and, together with GHPV, finch polyomavirus (FPyV), and canary polyomavirus (CaPyV) cause inflammation, swelling, and hemorrhage of the internal organs and skin. Polyomavirus infected birds show poor body conditions, and suffer from diarrhea and neurological signs (Bernáth and Szalai 1970; Bernier et al. 1981; Bozeman et al. 1981; Johne and Müller 1998, 2007; Phalen et al. 1997).

Polyomaviruses are small, non-enveloped viruses with an icosahedral capsid that encloses a circular dsDNA genome of 3.9–7.4 kbp in size (Moens et al. 2017). The polyomaviral early genes encode the large and small tumor antigens (LTA and STA) that are expressed before the onset of the viral DNA replication. The LTA and STA regulate the replication of the viral DNA and protein expression (Johne and Müller 2007; Kaszab et al. 2021b; Moens et al. 2017). The capsid forming viral proteins (VP1, VP2, and VP3) are encoded by late genes (Johne et al. 2007; Kaszab et al. 2021a, b; Moens et al. 2017). In addition to the essential genes, the polyomaviral genomes contain open reading frames (ORFs) whose existence vary among the viruses, such as the X or VP4 proteins of avian polyomaviruses. These ORFs are located in the same genomic regions, but the expressed proteins are not functionally homologues with each other (Johne et al. 2000, 2007; Johne and Müller 2001, 2007; Kaszab et al. 2021a; Moens et al. 2017).

In this study kidney, liver, and spleen specimens of 18 succumbed wild birds were collected in 2020 at the rescue station of Zoo and Botanical Garden, Budapest, Hungary. The birds were kept individually separated until their death. Experiments with or sampling from live animals were not performed. All methods were carried out in accordance with relevant guidelines and regulations. Ethical review and approval was not required for the study in accordance with the local legislation and institutional requirements. Sample lysis, nucleic acid extraction, and polyomavirus VP1 specific broad-spectrum nested PCR was carried out as described elsewhere (Fehér et al. 2022; Johne et al. 2005).

A highly divergent polyomavirus was identified from kidney and liver specimens of a common kestrel (*Falco tinnunculus*) that was transported to a rescue station with wing injury. Unfortunately, gross pathological data were not available for the succumbed bird. The complete genome of the kestrel polyomavirus (kesPyV) was amplified from the liver sample with back-to-back PCR primers designed based on the sequence of the diagnostic amplicons. The PCR mixture of 25 μL contained 1× Phusion Green Buffer, 0.3U Phusion DNA Polymerase (Thermo Fisher Scientific, Waltham, MA, USA), 200 μM dNTP mix, 200 nM of each primer (F1 primer: 5’-TTGTCACCGACCAACAAAGCC-3’; R1 primer 5’-ATCCCACAATATCACATGCTGACAC-3’) and 1 μL of the purified nucleic acid. The cycling protocol consisted of the steps: denaturation at 98°C for 30 s; 40 amplification cycles of 98°C for 10 s, 60°C for 30 s and 72°C for 3 min; a final extension step at 72°C for 5 min. The ~5000 bp amplicon was subjected to next-generation sequencing. DNA library was prepared with Illumina Nextera XT DNA Library Preparation Kit and Nextera XT Index Kit (Illumina, San Diego, CA, USA) as previously described, and was loaded onto iSeq100 sequencer flow cell and sequenced on an Illumina iSeq100 sequencer (Illumina, San Diego, CA, USA) (Olasz et al. 2019). The sequence reads were *de novo* assembled using the Genious Prime software v2022.2.2. The sequences were edited with the AliView software and were aligned with the MUSCLE algorithm implemented in the Geneious Prime software (Larsson 2014). MEGA X was used for determination of pairwise identity values (Kumar et al. 2018).

Altogether, 379,783 sequence reads assembled into the kesPyV genome *de novo* with a mean sequencing depth of 7,294x (75–9,890). The less represented priming sites were combined and covered by the sequence obtained by direct sequencing of the diagnostic PCR amplicon. The genome length of the novel kesPyV (GenBank acc. no. OQ540584) was 5025 nucleotide (nt) and contained the essential polyomaviral ORFs (LTA, STA, VP1, VP2 and VP3) as predicted with the ORF Finder tool (https://www.ncbi.nlm.nih.gov/orffinder/) (Fig. 1, Table 1) (Calvignac-Spencer et al. 2016; Ehlers and Moens 2014; Ehlers et al. 2019; Johne and Müller 2007; Kaszab et al. 2021a, b; Moens et al. 2017).

**Fig. 1:**
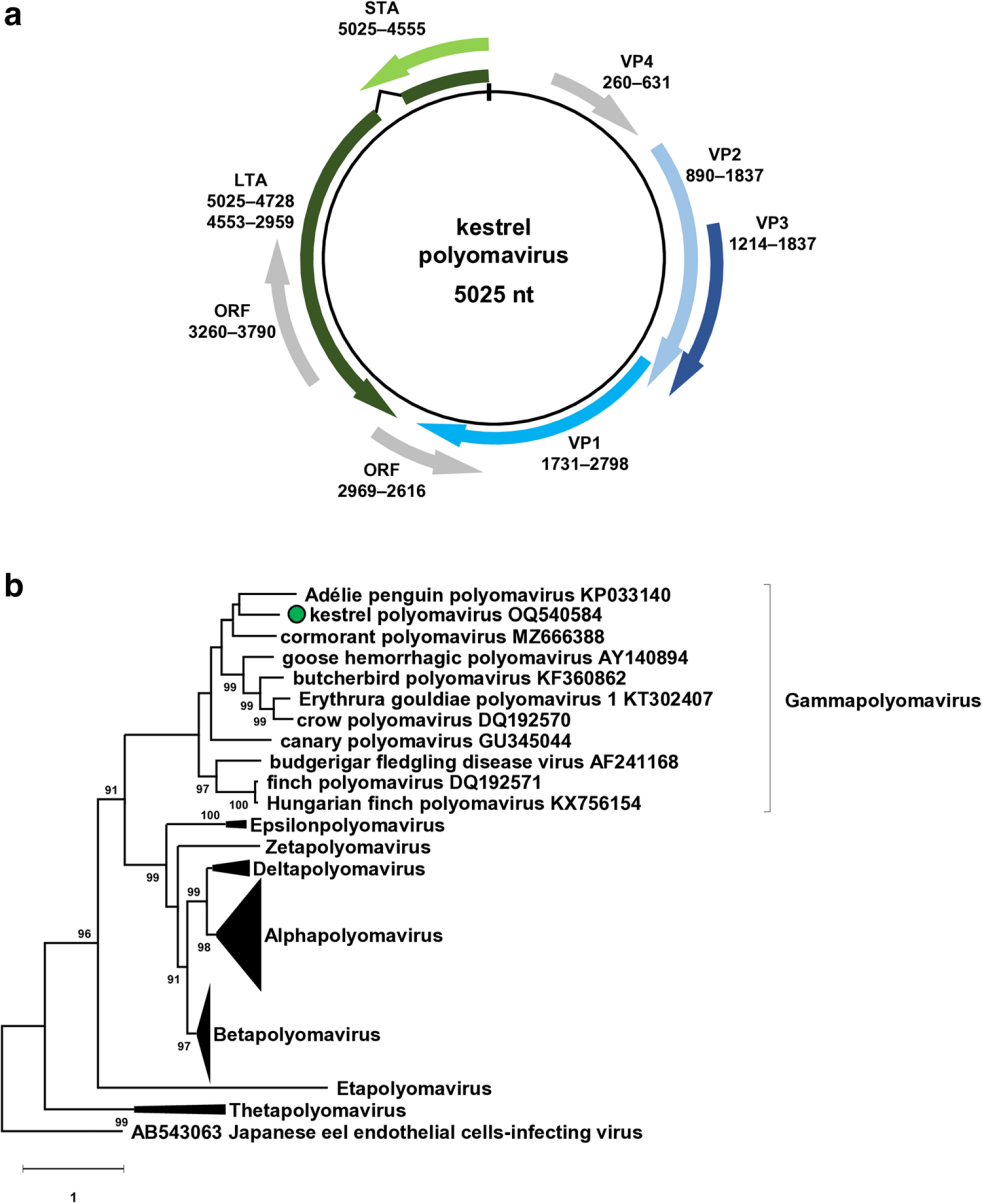
**(a)** Genomic organization of the kestrel polyomavirus (kesPyV) characterized in this study. Grey arrows represent predicted non-essential polyomaviral open reading frames (ORFs). **(b)** Phylogenetic tree of large T antigen aa sequences retrieved from the kesPyV genome and from the GenBank. The maximum likelihood tree was generated with the PhyML software, LG+G+I model, and aLRT SH-like branch support. Branch supports <80 were hidden. The root for the tree was set for the sequence of the Japaneese eel endothelial cells-infecting virus. kesPyV is labelled with green circle

**Table 1:** Characteristics of the predicted open reading frames (ORFs) identified in the kestrel polyomavirus genome. Nucleotide 1 of the genome is positioned as the first nucleotide upstream of the ORFs encoding the large and small tumor antigens (LTA and STA)

	Location nt	ORF length	
		nt	aa
LTA	5025–4728, 4553–2959	1893	630
STA	5025–4555	471	156
VP1	1731–2798	1068	355
VP2	890–1837	948	315
VP3	1214–1837	624	207
VP4	260–631	372	123
ORF	2969–2616	354	117
ORF	3260–3790	531	176

The deduced protein sequence of the VP3 started in-frame of the VP2 with the motif MALVPY that corresponded to the motif MALXXΦ (Φ= W, F, Y) determined for polyomaviruses (Ehlers and Moens 2014; Fehér et al. 2022). The LTA protein may be generated with alternative splicing and the aa sequence contained polyomaviral LTA-specific motifs, such as a conserved region motif FSELL (modified L to F), the hexapeptide HPDKGG between the second and third α-helix of the putative J domain, the pRB1-binding motif LYCSE, as well as the ATPase motifs GPVNTGKT and GSVPVNLE. Similarly to the LTA of other gammapolyomaviruses, the kesPyV LTA sequence contained a motif (CEDCKSQLDNATLRERKRKWMGGHIDDH; CX_2_CX_19_HX_3_H) resembling the zinc finger motif of mammalian polyomaviruses (CX_2_CX_7_HX_3_H, CX_2_CX_7_HX_2_H), but differing in length and aa composition from the mammalian variants (Ehlers and Moens 2014; Fehér et al. 2022). In contrast to mammalian polyomaviruses, the LTA of gammapolyomaviruses and kesPyV had a low rate of positively charged residues in the conserved nuclear localization signal.

The LTA, STA, VP1, VP2 and VP3 of the kesPyV represented up to 54.7–65.2% nt and 45.2–67.5% aa pairwise identities with the homologous sequences of the cormorant polyomavirus (CoPyV), BFDV, GHPV, and Adélie penguin polyomavirus. The LTA of the kesPyV, used for determination of relations among PyVs, shared 59.2% nt and 57.3% aa pairwise identity with its closest relative, the LTA of CoPyV (Fehér et al. 2022). These values are below the 15% cut-off value set for species demarcation within the *Polyomaviridae* family (Calvignac-Spencer et al. 2016). Maximum likelihood phylogenetic analysis was performed with the PhyML software (LG+G+I model, aLRT SH-like branch support) using representative sequences of the polyomavirus species (Guindon et al. 2010). The kesPyV clustered with gammapolyomaviruses, but formed a well-separated branch in the phylogenetic tree (Fig. 1). The data confirmed that the kesPyV belongs to a novel species of the *Gammapolyomavirus* genus, tentatively named *Gammapolyomavirus faltin*.

Besides the essential genes, additional ORFs with either forward or reverse orientation were also identified in the kesPyV genome (Fig. 1, Table 1). Three of these were >303 nt in length potentially encoding proteins of >100 aa. Although the BLAST search did not reveal matches with any GenBank records, one of these had very characteristic Leu- and Pro-rich regions. The 372 nt long ORF located upstream of the VP2 at the coding region of the ORF-X and VP4 of other avian polyomaviruses. In contrast to the gammapolyomaviral ORF-X and VP4, this ORF was not interrupted with intron(s) according to the splicing site prediction tools Alternative Splice Site Predictor and the NetGene2 Server (Brunak et al. 1991; Wang and Marín 2006). In respect of some aa motifs, the deduced 123 aa long protein resembled the agnoprotein 2a and 2b of BFDV (hereinafter referred to as VP4-2a and VP4-2b). Moreover, this ORF shared greater similarity with the E4 gene of human papillomaviruses (HPVs, *Papillomaviridae* family). Therefore, this particular ORF of the kesPyV has been named VP4 (Fig. 1, Table 1). The overall arrangement and structure of the kesPyV VP4 aa modules represented the closest relationship to the E4 protein of HPV16 and HPV18 (Fig. 2 and Online Resource). The kesPyV VP4 shared <25% aa pairwise identity with the BFDV VP4-2a and VP4-2b (GenBank acc. no. KT203765), and ≤30% aa pairwise identity with E4 of HPV16 and HPV18 (GenBank acc. no. LC718898 and ON322746).

**Fig. 2:**
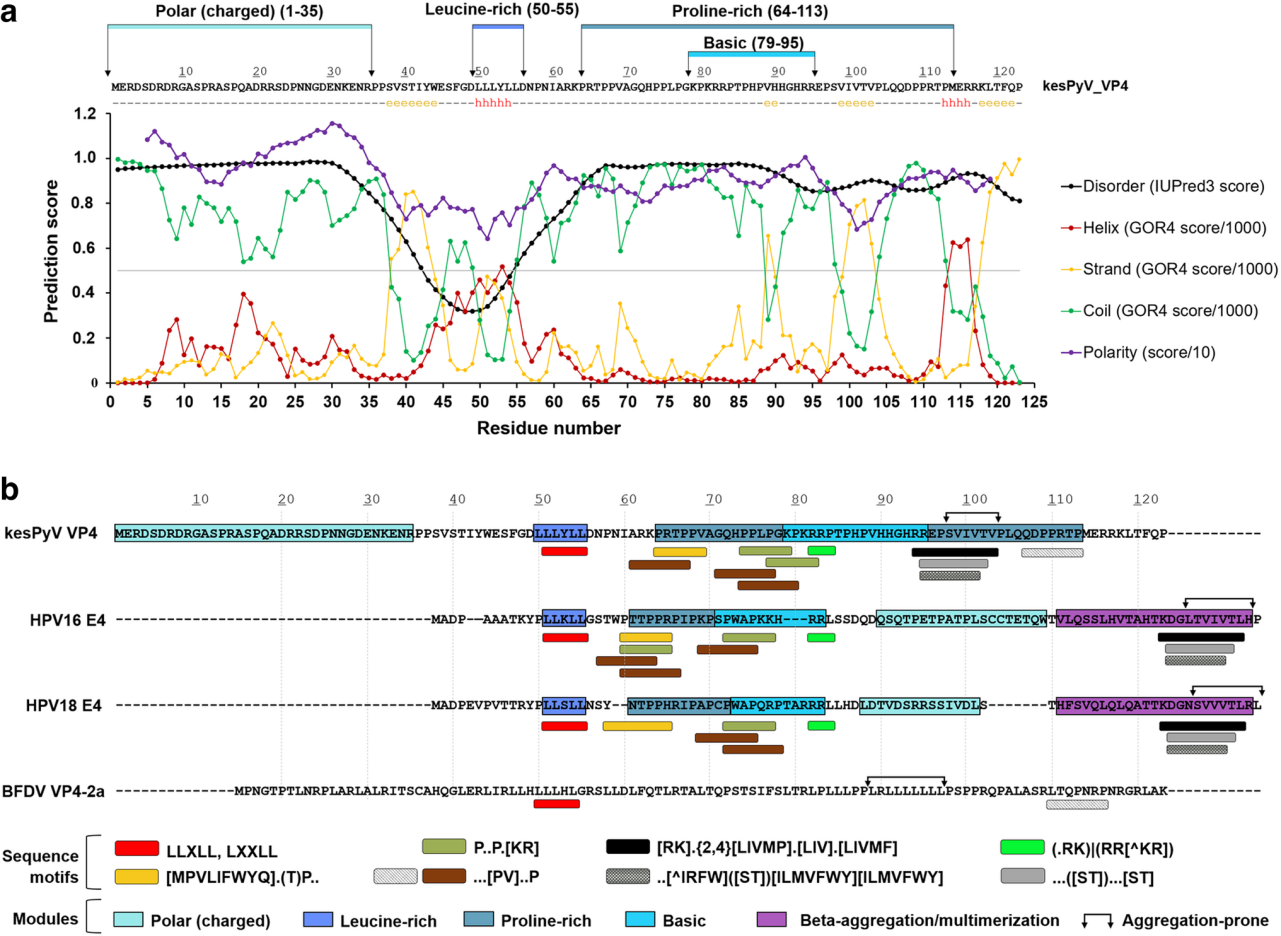
**(a)** Sequence and predicted structural characteristics of the kestrel polyomavirus (kesPyV) viral protein 4 (VP4). The predicted secondary structural arrangement is indicated below the sequence (“e”: strand, “h”: helix, “-“: coil). The propensities of secondary structural elements (predicted by GOR4) and disordered regions (predicted by IUpred3), as well as calculated polarity values (predicted by Expasy and ProtScale) are plotted in the graph. The polar, basic, Leu- and Pro-rich regions are indicated in the upper part of the figure. **(b)** Linear sequence motifs and modular motif organization within the kesPyV VP4, the E4 proteins of human papillomavirus (HPV) 16 and HPV18, as well as the budgerigar fledgling disease virus VP4-2a proteins. Eukaryotic linear motifs were selected based on the ELM database (see Online Resource). Regions predicted to be prone to aggregation and identified with the highest Aggrescan scores are labelled with arrows. Comparison with HPVs based on the study of Doorbar (2013)

Multiple species of BFDV VP4 are produced by splicing of late RNAs. The VP4 (VP4-1a, 176 aa) and VP4Δ (VP4-1b, 112 aa) are produced from the same ORF and these proteins have role in virus replication and release of progeny viruses. Both proteins induce apoptosis of the infected cells and may inhibit IFN-β expression (Johne et al. 2000, 2007; Johne and Müller 2007; Ma et al. 2019). The BFDV VP4-1a interacts with the viral DNA and the major viral proteins, and has a scaffolding function incorporating into the viral particles (Johne and Müller 2001; Johne et al. 2007). The BFDV VP4-2a (109 aa) and VP4-2b (79 aa) originate from a distinct ORF of BFDV, but no detailed information is available about their properties and function (Luo et al. 1995). Likewise, variable papillomaviral E4 forms have been described that are generated by RNA splicing and post-translationally by proteases (Doorbar 2013). Although low probability of splicing was predicted in the kesPyV VP4, numerous protease cleavage sites (details not shown) were identified implying that more than one protein species are produced by post-translational ways.

The main features of the translated full length proteins were estimated by using sequence-based *in silico* approaches. The protein features were calculated using tools available at the Expasy web server (e.g. polarity by the ProtScale module) (Gasteiger et al. 2005). The functional sites were predicted by the ELM resource (Eukaryotic Linear Motif resource for Functional Sites in Proteins) using default parameters (Kumar et al. 2022). The kesPyV VP4 showed a characteristic distribution of polar and apolar residues. The N-terminal region (aa 1-35) was found to be highly polar and rich in Arg, Asp, Glu and Ser residues, followed by a region with a Leu-rich motif (^50^LLLYLL^55^). The downstream region (aa64-113) contains multiple Pro with basic residues in its central part (aa79-95) (Fig. 2a). Prediction of the secondary structural arrangement by GOR4 web server revealed that the main part of the protein is not folded into locally structured elements (Combet et al. 2000). The central region and the C-terminus is ordered and is folded into short β-strands, while a short region with the Leu-rich motif has an α-helical structure (Fig. 2a). In agreement with the low overall propensity for structurally ordered elements, the disorder prediction (long disorder, IUPred3 web server) implied that the protein is mainly disordered, and the region with the lowest disorder propensity is in the proximity of the Leu-rich motif (Erdős et al. 2021). Due to the presence of multiple Pro residues, the C-terminal region was not predicted to be globular (Fig. 2a). We attempted to estimate the three-dimensional structure as well. A template search, performed by using the SWISS-MODEL automated protein structure homology modeling web server, showed a maximum of 30% sequence identity with the potential templates at only a low sequence coverage (0.33%), therefore, it was not possible to model the tertiary structure of the protein reliably (Waterhouse et al. 2018).

The N-terminal region of the full-length papillomaviral E4 contains an α-helix with LLXLL Leu cluster. This motif and the upstream residues are thought to be responsible for cytokeratin association that is facilitated by phosphorylation of Ser/Thr sites embedded in the central region of the protein rich in Pro and basic residues (Arg, Lys) (Fig. 2 and Online Resource) (Doorbar 2013). Variable kinases, including mitogen-activated protein kinases (MAPK) and cyclin-dependent kinases (CDK), regulate E4 transformation and cellular destruction through these sites. Interaction with the cellular keratin network enhances papillomavirus escape from the cells (Doorbar 2013). Both the LLXLL cluster within a probable α-helix (aa50-55), as well as the Pro and Arg rich region with Ser/Thr residues (aa64-95) and kinase docking motifs can be recognized in the kesPyV VP4 (Fig. 2 and Online Resource).

The C-terminal beta-aggregation motif is responsible for large-scale self-multimerization after cleavage of the N-terminal keratin-binding motif of the papillomaviral E4. A region characterized by negatively charged aa (Asp, Glu), Pro and Thr is positioned between this beta-aggregation motif and the above mentioned Pro- and Arg-rich region of the E4 (Fig. 2) (Doorbar 2013). As for HPVs, high probability for amyloid formation has been calculated for the aa98-103 region of the kesPyV VP4 by the AGGRESCAN and the AMYPred-FRL online tools implying aggregation-prone nature of the C-terminus of the Pro-rich module (Charoenkwan et al. 2022; Conchillo-Solé et al. 2007). Likewise, predisposition for aggregation was detected in the Leu-rich region of the HPV E4, kesPyV VP4 and BFDV VP4-2a but with lower probability scores than that of calculated for C-termini. As compared to the papillomavirus E4, the potential multimerization site and the Pro- and Arg-rich central region is linked with a shorter stretch in the kesPyV VP4. The C-terminal region of the kesPyV VP4 (aa104-123) encompasses negatively charged aa, Pro, Thr and Arg residues downstream of the beta-aggregation motif, while the N-terminal region is densely interspersed with negatively and positively charged, as well as Ser residues (Fig. 2). A similar N-terminal and C-terminal region is missing from the papillomaviral E4.

Production of the papillomaviral E4 is associated with viral amplification thus this molecule could be a biomarker to follow progression of the infection (Griffin et al. 2015). HPV E4 accumulation has been observed at the start of excessive viral genome replication at S/G2 cell cycle transition; the post-translationally modified E4 species can support CDK sequestration, which events lead to G2 cycle arrest (Doorbar 2013). Although the importance of the existing CDK binding sites is unknown, kesPyV VP4 may have E4-like cell cycle modification properties.

In addition to the VP4, a putative non-essential ORF were identified in the kesPyV genome in forward direction. The ORF (nt 3260-3790) corresponded in location to ORFs encoding >100 aa long proteins in the genome of the GHPV, CaPyV, CoPyV, Hungarian finch polyomavirus and corvus polyomavirus. As these ORFs located in the coding region of the LTA, the sequence of the kesPyV shared unsurprisingly higher pairwise aa identity (65.1%) with the references than unknown ORFs in other genomic regions. The ORF nt 2969-2616 showed reverse orientation and overlapped the 3’ LTA and 3’ VP1 of the kesPyV genome. ORFs potentially encoding proteins ≥80 aa were identified in the same genomic region of the GHPV, CaPyV and FPyV genomes, with pairwise aa identities ≤39.7%. A comprehensive study would help clarify what type of RNAs are edited from the pre-mRNAs of the avian polyomaviruses, but the lack of routine culturing protocols reduces the chance to carry out such investigations.

Some recent viruses have been described with genomic properties of both papillomaviruses and polyomaviruses. A large part of the ~7.3 kb long bandicoot papillomatosis carcinomatosis virus type 1 and type 2 (BPCV1 and BPCV2) genome has been shown close relationship with the papillomaviral capsid protein encoding L1 and L2 late ORFs. The smaller parts (~2300 nt) of the BPCV genomes have been found to contain ORFs in an opposite orientation in the complementary DNA strand that encode putative early proteins resembling of the polyomaviral LTA and STA (Bennett et al. 2008; Woolford et al. 2007). These ‘hybrid’ viruses may have emerged by recombination or may be descendants of common ’papovavirus’ ancestors (Bennett et al. 2008; Woolford et al. 2007). Although there are no unambiguous traces of similar events, and recombination could not be detected among kesPyV and HPV sequences (data not shown), the results presented here draw attention to presumably homologous functions of kesPyV VP4 and HPV E4. The predicted similarities of the proteins promote characterization of the viruses and aid the design of experiments needed to reveal virus-host interactions.

Compared to mammalian polyomaviruses, avian polyomaviruses have broader host-spectrum and occur in farmed, pet, and wild birds that favours viral spread via animal transport and migration (Circella et al. 2017; Johne and Müller 1998; Wang et al. 2022). To avoid economic losses and threats of avian species conservation more attention should be paid to understanding of avian polyomaviruses to set up an effective protection against them.

### Supplementary Information

ESM 1 (DOCX 17 kb)

## Data Availability

The datasets generated for this study can be found in the GenBank with accession number OQ540584.

## References

[CR1] Bennett MD, Woolford L, Stevens H, Van Ranst M, Oldfield T, Slaven M, O'Hara AJ, Warren KS, Nicholls PK (2008). Genomic characterization of a novel virus found in papillomatous lesions from a southern brown bandicoot (*Isoodon obesulus*) in Western Australia. Virology.

[CR2] Bernáth S, Szalai F (1970). Investigation for clearing the etiology of the disease appeared among goslings in 1969 (in Hungarian). Magyar Állatorvosok Lapja.

[CR3] Bernier G, Morin M, Marsolais G (1981). A generalized inclusion body disease in the budgerigar (*Melopsittacus undulatus*) caused by a papovavirus-like agent. Avian Dis.

[CR4] Bozeman LH, Davis RB, Gaudry D, Lukert PD, Fletcher OJ, Dykstra MJ (1981). Characterization of a papovavirus isolated from fledgling budgerigars. Avian Dis.

[CR5] Brunak S, Engelbrecht J, Knudsen S (1991). Prediction of Human mRNA Donor and Acceptor Sites from the DNA Sequence. J Mol Biol.

[CR6] Calvignac-Spencer S, Feltkamp MCW, Daugherty MD, Moens U, Ramqvist T, Johne R, Ehlers B (2016). A taxonomy update for the family Polyomaviridae. Arch Virol.

[CR7] Charoenkwan P, Ahmed S, Nantasenamat C, Quinn JMW, Moni MA, Lio' P, Shoombuatong W (2022). AMYPred-FRL is a novel approach for accurate prediction of amyloid proteins by using feature representation learning. Sci Rep.

[CR8] Circella E, Caroli A, Marino M, Legretto M, Pugliese N, Bozzo G, Cocciolo G, Dibari D, Camarda A (2017). Polyomavirus Infection in Gouldian Finches (*Erythrura gouldiae*) and Other Pet Birds of the Family Estrildidae. J Comp Pathol.

[CR9] Combet C, Blanchet C, Geourjon C, Deléage G (2000). NPS@: network protein sequence analysis. Trends Biochem Sci.

[CR10] Conchillo-Solé O, de Groot NS, Avilés FX, Vendrell J, Daura X, Ventura S (2007). AGGRESCAN: a server for the prediction and evaluation of "hot spots" of aggregation in polypeptides. BMC Bioinformatics.

[CR11] Doorbar J (2013). The E4 protein; structure, function and patterns of expression. Virology.

[CR12] Ehlers B, Moens U (2014). Genome analysis of non-human primate polyomaviruses. Infect Genet Evol.

[CR13] Ehlers B, Anoh AE, Ben Salem N, Broll S, Couacy-Hymann E, Fischer D, Gedvilaite A, Ingenhütt N, Liebmann S, Martin M, Mossoun A, Mugisha L, Muyembe-Tamfum JJ, Pauly M, Pérez de Val B, Preugschas H, Richter D, Schubert G, Szentiks CA, Teichmann T, Walter C, Ulrich RG, Wiersma L, Leendertz FH, Calvignac-Spencer S (2019). Novel Polyomaviruses in Mammals from Multiple Orders and Reassessment of Polyomavirus Evolution and Taxonomy. Viruses.

[CR14] Erdős G, Pajkos M, Dosztányi Z (2021). IUPred3: prediction of protein disorder enhanced with unambiguous experimental annotation and visualization of evolutionary conservation. Nucleic Acids Res.

[CR15] Fehér E, Kaszab E, Bali K, Hoitsy M, Sós E, Bányai K (2022). A novel gammapolyomavirus in a great cormorant (*Phalacrocorax carbo*). Arch Virol.

[CR16] Gasteiger E, Hoogland C, Gattiker A, Duvaud S, Wilkins MR, Appel RD, Bairoch A (2005) Protein Identification and Analysis Tools on the Expasy Server. In: Walker JM editor: The Proteomics Protocols Handbook. Humana Press pp 571–607

[CR17] Griffin H, Soneji Y, Van Baars R, Arora R, Jenkins D, van de Sandt M, Wu Z, Quint W, Jach R, Okon K, Huras H, Singer A, Doorbar J (2015). Stratification of HPV-induced cervical pathology using the virally encoded molecular marker E4 in combination with p16 or MCM. Mod Pathol.

[CR18] Guindon S, Dufayard JF, Lefort V, Anisimova M, Hordijk W, Gascuel O (2010). New Algorithms and Methods to Estimate Maximum-Likelihood Phylogenies: Assessing the Performance of PhyML 3.0. Syst Biol.

[CR19] Johne R, Müller H (1998). Avian polymavirus in wild birds: genome analysis of isolates from Falconiformes and Psittaciformes. Arch Virol.

[CR20] Johne R, Müller H (2001). Avian polyomavirus agnoprotein 1a is incorporated into the virus particle as a fourth structural protein, VP4. J Gen Virol.

[CR21] Johne R, Müller H (2007). Polyomaviruses of birds: etiologic agents of inflammatory diseases in a tumor virus family. J Virol.

[CR22] Johne R, Jungmann A, Müller H (2000). Agnoprotein 1a and agnoprotein 1b of avian polyomavirus are apoptotic inducers. J Gen Virol.

[CR23] Johne R, Enderlein D, Nieper H, Müller H (2005). Novel polyomavirus detected in the feces of a chimpanzee by nested broad-spectrum PCR. J Virol.

[CR24] Johne R, Paul G, Enderlein D, Stahl T, Grund C, Müller H (2007). Avian polyomavirus mutants with deletions in the VP4-encoding region show deficiencies in capsid assembly and virus release, and have reduced infectivity in chicken. J. Gen. Virol.

[CR25] Kaszab E, Marton S, Erdélyi K, Bányai K, Fehér E (2021). Genomic evolution of avian polyomaviruses with a focus on budgerigar fledgling disease virus. Infect Genet Evol.

[CR26] Kaszab E, Szabadi L, Kepner A, Bajnóczi P, Lengyel G, Bányai K, Fehér E (2021). Viral gene expression profile of goose haemorrhagic polyomavirus in susceptible primary cell. Avian Pathol.

[CR27] Kumar S, Stecher G, Li M, Knyaz C, Tamura K (2018). MEGA X: Molecular Evolutionary Genetics Analysis across computing platforms. Mol Biol Evol.

[CR28] Kumar M, Michael S, Alvarado-Valverde J, Mészáros B, Sámano-Sánchez H, Zeke A, Dobson L, Lazar T, Örd M, Nagpal A, Farahi N, Käser M, Kraleti R, Davey NE, Pancsa R, Chemes LB, Gibson TJ (2022). The Eukaryotic Linear Motif resource: 2022 release. Nucleic Acids Res.

[CR29] Larsson A (2014). AliView: a fast and lightweight alignment viewer and editor for large datasets. Bioinformatics.

[CR30] Luo D, Müller H, Tang XB, Hobom G (1995). Early and late pre-mRNA processing of budgerigar fledgling disease virus 1: identification of viral RNA 5' and 3' ends and internal splice junctions. J Gen Virol.

[CR31] Ma J, Wu R, Tian Y, Zhang M, Wang W, Li Y, Tian F, Cheng Y, Yan Y, Sun J (2019). Isolation and characterization of an Aves polyomavirus 1 from diseased budgerigars in China. Vet Microbiol.

[CR32] Moens U, Krumbholz A, Ehlers B, Zell R, Johne R, Calvignac-Spencer S, Lauber C (2017). Biology, evolution, and medical importance of polyomaviruses: An update. Infect Genet Evol.

[CR33] Olasz F, Mészáros I, Marton S, Kaján GL, Tamás V, Locsmándi G, Magyar T, Bálint Á, Bányai K, Zádori Z (2019). A Simple Method for Sample Preparation to Facilitate Efficient Whole-Genome Sequencing of African Swine Fever Virus. Viruses.

[CR34] Phalen DN, Wilson VG, Graham DL (1997). Prevalence of neutralizing antibody and virus shedding in psittacine birds infected with avian polyomavirus. J Avian Med Surg.

[CR35] Schmidlin K, Kraberger S, Cook C, DeNardo DF, Fontenele RS, Van Doorslaer K, Martin DP, Buck CB, Varsani A (2021). A novel lineage of polyomaviruses identified in bark scorpions. Virology.

[CR36] Wang M, Marín A (2006). Characterization and Prediction of Alternative Splice Sites. Gene.

[CR37] Wang CW, Chen YL, Mao SJT, Lin TC, Wu CW, Thongchan D, Wang CY, Wu HY (2022). Pathogenicity of Avian Polyomaviruses and Prospect of Vaccine Development. Viruses.

[CR38] Waterhouse A, Bertoni M, Bienert S, Studer G, Tauriello G, Gumienny R, Heer FT, de Beer TAP, Rempfer C, Bordoli L, Lepore R, Schwede T (2018). SWISS-MODEL: homology modelling of protein structures and complexes. Nucleic Acids Res.

[CR39] Woolford L, Rector A, Van Ranst M, Ducki A, Bennett MD, Nicholls PK, Warren KS, Swan RA, Wilcox GE, O'Hara AJ (2007). A novel virus detected in papillomas and carcinomas of the endangered western barred bandicoot (Perameles bougainville) exhibits genomic features of both the Papillomaviridae and Polyomaviridae. J Virol.

