# Supplementary material for: Structural similarity of human papillomavirus E4 and polyomaviral VP4 exhibited by genomic analysis of the common kestrel (Falco tinnunculus) polyomavirus
Source: Vet Res Commun. 2023 Sep 9;48(1):309–15. doi: 10.1007/s11259-023-10210-1 (PMC10810995; doi:10.1007/s11259-023-10210-1)
Supplement: Supplementary file 1 — (DOCX 17 kb) [file 11259_2023_10210_MOESM1_ESM.docx]

**Structural similarity of human papillomavirus E4 and polyomaviral VP4 exhibited by genomic analysis of the common kestrel (*Falco tinnunculus*) polyomavirus**

**Veterinary Research Communications**

**Enikő Fehér^1,2,3*^, Eszter Kaszab^1,2,4^, János András Mótyán^5^, Dóra Máté^1^, Krisztina Bali^1,2^, Márton Hoitsy^6,7^, Endre Sós^6,7^, Ferenc Jakab^3^, Krisztián Bányai^1,2,8^**

^1^ HUN-REN Veterinary Medical Research Institute, Budapest, Hungary

^2^ National Laboratory for Infectious Animal Diseases, Antimicrobial Resistance, Veterinary Public Health and Food Chain Safety, Budapest, Hungary

^3^ National Laboratory of Virology, Szentágothai Research Centre, University of Pécs, Pécs, Hungary

^4^ Institute of Metagenomics, University of Debrecen, Debrecen, Hungary

^5^ Department of Biochemistry and Molecular Biology, Faculty of Medicine, University of Debrecen, Debrecen, Hungary

^6^ Conservation and Veterinary Services, Budapest Zoo and Botanical Garden, Budapest, Hungary

^7^ Department of Exotic Animal and Wildlife Medicine, University of Veterinary Medicine, Budapest, Hungary

^8^ Department of Pharmacology and Toxicology, University of Veterinary Medicine, Budapest, Hungary

**Correspondence:**

Enikő Fehér

[feher.eniko@vmri.hun-ren.hu](mailto:feher.eniko@vmri.hun-ren.hu)

**Table 1.** Linear aa sequence motifs identified in viral protein 4 (VP4) protein of kestrel polyomavirus (kesPyV), VP4-2a (agnoprotein 2a) of beak and feather disease virus (BFDV), and the E4 of human papillomavirus 16 and 18 (HPV16 and HPV18). Motifs with similar location within the proteins of different viruses are written in black, while those with distinct location are in grey

| **Identified motif and function** | **kesPyV VP4** | **BFDV VP4 2a** | **HPV16** | **HPV18** |
| --- | --- | --- | --- | --- |
| **LLXLL: cytokeratin association;**  **LXXLL: nuclear receptor box motif** | **^51^LLYLL^55^** | **^33^LLHLL^37^** | **^12^LLKLL^16^** | **^14^LLSLL^18^** |
| **[MPVLIFWYQ].(T)P..**  **Mediates docking of CDK substrates and regulators to cyclin-CDK-bound Cks1** | **^64^PRTPPV^69^**  **^110^PRTPME^115^** | **not found** | **^21^PTTPPR^26^**  **^52^PETPAT^57^**  **^55^PATPLS^56^** | **^21^YNTPPH^26^** |
| **P..P.[KR]**  **Recognized by class II SH3 domains** | **^74^PPLPGK^79^**  **^77^PGKPKR^82^** | **^99^PNRPNR^104^** | **^21^PTTPPR^26^**  **^33^PWAPKK^38^** | **^33^PWAPQR^38^** |
| **...[PV]..P**  **Recognized by class I SH3 domains** | **^61^ARKPRTP^67^**  **^71^GQHPPLP^77^**  **^74^PPLPGKP^80^** | **not found** | **^18^STWPTTP^24^**  **^21^ PTTPPRP^27^**  **^30^KPSPWAP^36^** | **^30^APCPWAP^36^**  **^33^PWAPQRP^39^** |
|  | **not found** | **^81^LLLPSPP^87^**  **^84^PSPPRQP^90^** | **^49^SQTPETP^55^**  **^52^PETPATP^58^** | **not found** |
|  | **^107^QDPPRTP^113^** | **^96^LTQPNRP^102^** | **not found** | **not found** |
| **...([ST])P..**  **Pro-directed kinase (e.g. MAPK) phosphorylation site** | **^9^RGASPRA^15^**  **^13^PRASPQA^19^**  **^63^KPRTPPV^69^**  **^82^RRPTPHP^88^**  **^109^PPRTPME^115^** | **^2^PNGTPTL^8^**  **^82^LLPSPPR^88^** | **^20^WPTTPPR^26^**  **^29^PKPSPWA^35^**  **^48^QSQTPET^54^**  **^51^TPETPAT^57^**  **^54^TPATPLS^60^** | **^20^SYNTPPH^26^** |
| **[RK].{2,4}[LIVMP].[LIV].[LIVMF]**  **Kinase docking motif mediating interaction to ERK1/2 and p38 subfamilies of MAP kinases.** | **^94^RREPSVIVTV^103^** | **^10^RPLARLALRI^19^**  **^29^RLIRLLHLLL^38^**  **^32^RLLHLLLHL^40^**  **^76^RLLLLLLL^83^** | **^81^KDGLTVIVTL^89^** | **^77^KDGNSVVVTL^86^** |
| **(.RK)\|(RR[^KR])**  **N-Arg dibasic convertase (NRD/Nardilysin) cleavage site (X-\|-R-K or R-\|-R-X).** | **^21^RRS^23^**  **^61^ARK^63^**  **^82^RRP^84^**  **^94^RRE^96^**  **^116^RRK^118^** | **not found** | **^40^RRL^42^** | **^43^RRL^45^**  **^55^RRS^57^** |
| **([LIVMFYWPR]R[^YFWDE]{0,1}R)\|(R[^YFWDE]{0,1}R[LIVMFYWPR])**  **Di-Arg ER retention motif** | **^82^RRP^84^** | **^104^RGRL^107^** | **^40^RRL^42^** | **^42^RRR^44^**  **^43^RRL^45^** |
| **...([ST])...[ST]**  **GSK3 phosphorylation recognition site** | **^9^RGASPRAS^16^**  **^95^REPSVIVT^102^** | **^53^TALTQPST^60^**  **^57^QPSTSIFS^64^** | **^15^LLGSTWPT^22^**  **^16^LGSTWPTT^23^**  **^65^TQWTVLQS^72^**  **^70^LQSSLHVT^77^**  **^89^DGLTVIVT^89^** | **^13^PLLSLLNS^20^**  **^51^TVDSRRSS^58^**  **^60^VDLSTHFS^67^**  **^78^DGNSVVVT^85^** |
| **..[^IRFW]([ST])[ILMVFWY][ILMVFWY]. S/T residue phosphorylated by Plk4** | **^38^SVSTIYW^44^**  **^95^REPSVIV^101^** | **^58^PSTSIFS^64^** | **^82^DGLTVIV^88^** | **^13^PLLSLLN^19^**  **^55^RRSSIVD^61^**  **^78^DGNSVVV^84^** |
| **..(T)..[ILV].**  **Phosphothreonine motif binding FHA domains with preference for large aliphatic aa at pT+3 position** | **^64^PRTPPVA^70^**  **^100^IVTVPLQ^106^** | **^3^NGTPTLN^9^** | **^83^GLTVIVT^89^** | **not found** |
